# Increased Risk of Pulmonary Tuberculosis in Patients with Depression: A Cohort Study in Taiwan

**DOI:** 10.3389/fpsyt.2017.00235

**Published:** 2017-11-13

**Authors:** Kao-Chi Cheng, Kuan-Fu Liao, Cheng-Li Lin, Shih-Wei Lai

**Affiliations:** ^1^College of Medicine, China Medical University, Taichung, Taiwan; ^2^Department of Family Medicine, China Medical University Hospital, Taichung, Taiwan; ^3^Department of Internal Medicine, Taichung Tzu Chi General Hospital, Taichung, Taiwan; ^4^College of Medicine, Tzu Chi University, Hualien, Taiwan; ^5^Graduate Institute of Integrated Medicine, China Medical University, Taichung, Taiwan; ^6^Management Office for Health Data, China Medical University Hospital, Taichung, Taiwan

**Keywords:** epidemiology, cohort study, depression, pulmonary tuberculosis, comorbidities

## Abstract

**Background/objective:**

Tuberculosis (TB) and depression were major public health issues worldwide and the mutual causative relationships between them were not exhaustive. This study was performed to explore the association between depression, comorbidities, and the risk of pulmonary TB in Taiwan.

**Methods:**

The cohort study used the database of the Taiwan National Health Insurance Program. The depression group included 34,765 subjects aged 20–84 years with newly diagnosed depression from 2000 to 2012, and the non-depression group included 138,187 randomly selected subjects without depression. Both depression and non-depression groups were matched with respect to sex, age, and comorbidities. We explored the incidence of pulmonary TB at the end of 2013 in both the groups and used multivariable Cox proportional hazards regression model to explore the hazard ratio (HR) and 95% confidence interval (CI) for the risk of pulmonary TB associated with depression.

**Results:**

The overall incidence of pulmonary TB was 1.16-fold greater in the depression group than that in the non-depression group (1.52 vs. 1.31 per 1,000 person-years, 95% CI 1.12, 1.21). The multivariable Cox proportional hazards regression analysis revealed that the adjusted HR of pulmonary TB was 1.15 for the depression group (95% CI 1.03, 1.28), compared with the non-depression group.

**Conclusion:**

Depression is associated with 1.15-fold increased hazard of pulmonary TB in Taiwan.

## Introduction

Tuberculosis (TB) is a major public health issue with a relatively high incidence and prevalence. It is also a chronic infectious multi-systemic disease which has burdened the socioeconomic and health-care aspect in the past few decades. According to the World Health Organization (WHO) report, at least 10.4 million people demonstrated new incidents of TB worldwide in 2014 (1). Half of all the global new cases of TB occurred in some developing Asian countries, such as Bangladesh, China, India, and Pakistan (2). In Taiwan, the annual incidence was 63.7 and 53 cases per 100,000 person-years in 2006 and 2012 (3, 4). Meanwhile, we observed the declined trend of TB incidence over the past 50 years in Taiwan after efforts of Taiwan Centers for Disease Control (CDC) (5). All efforts for recent decades decreasing incidence of TB in Taiwan by Taiwan CDC, including directly observed treatment, short course (DOTS) for sputum smear-positive patients, DOTS-plus strategy for multidrug resistance tuberculosis patients and profound treatment for latent TB children. After all control measurements mentioned above, in 2013 alone, there were 54.5 new diagnosed cases and 2.8 deaths per 100,000 populations in Taiwan (6). The first-line medications for TB include rifampicin, isoniazid, pyrazinamide, and ethambutal, the treatment duration was suggested 6 months in general. Until 2009 in Taiwan, the treatment success rate is up to 87% (7).

In addition to developing countries, 4–6% of the population in United States have latent infections of TB (8). Previous studies have demonstrated multiple risk factors associated with pulmonary TB, including chronic obstructive pulmonary disease (COPD), pneumoconiosis (9, 10), chronic kidney disease (CKD), diabetes mellitus (DM), and human immunodeficiency virus (HIV) (11). Male gender, old age, tobacco smoking, and alcohol consumption have also been shown to be related with pulmonary TB (12, 13).

Depression is the second leading cause of disease burden, accounting for 3.8% of the total disability-adjusted life year in the year 2010, and it causes the largest amount of non-fatal burden which accounts for almost 8.2% of all total years lived with disability worldwide (14). At the same time, we found that the prevalence of depression ranged from 1.2 to 26% in Taiwan’s community studies in 2005 and in large scale, more 10 thousands respondents study (15, 16). Although 1.7% in the 1980s, the incidence rate of depression in elderly raised up to 19.7% gradually. Furthermore, adults 65 years and older accounts for 10.53% of all populations (17), will be a critical public issue in Taiwan.

Among the various organs and tissues in humans, lungs are predominantly affected by TB infection. Awareness about depression and its role in chronic disorders such as rheumatoid arthritis and COPD has increased over the years (18). Psychiatric illness may develop subsequent to TB infection, and mood disorders seem to be particularly common in TB populations compared to populations infected with other chronic diseases (6, 8, 19). Due to the frequent comorbidity of TB and associated mood disorders, particularly depression, it is very important for general physicians and psychiatric specialists to be mindful of depression while treating patients with TB. Although depression has been shown to be common in patients with TB (20–22), Up to this day, the mechanism and relationships between depression and pulmonary TB is complex and elusive. In the past few decades, to the best of our knowledge, no study as yet has examined the causative relationship between depression and pulmonary TB in Taiwan. Thus, we aimed to determine the presence of depression in patients suffering from pulmonary TB in Taiwan.

## Materials and Methods

### Design and Data Source

Taiwan is a sovereign and independent country with more than 23 million residents (23–34). This population-based cohort study used the database of the Taiwan National Health Insurance Program. This insurance program began in March 1, 1995, and it has covered about 99% of 23 million residents living in Taiwan (35). The details of the program have been well documented in previous studies (36–40). This study was approved by the Ethics Review Board of China Medical University in Taiwan (CMUH-104-REC2-115).

### Participants

We selected subjects aged 20–84 years with newly diagnosed depression from 2000 to 2012 into the depression group [the International Classification of Diseases (ICD) 9th Revision, ICD-9 codes 296.2, 296.3, 300.4, and 311]. To increase the statistical power, four subjects without depression were randomly selected, for each subject with depression, into the non-depression group. The index date was defined as the date depression was diagnosed. Both depression and non-depression groups were matched with respect to sex, age (every 5-year interval), and comorbidities.

### Comorbidities

The following comorbidities that could be potentially related to pulmonary TB were included: alcohol-related disease, asbestosis, CKD, COPD, DM, HIV infection, gastrectomy, pneumoconiosis, splenectomy, and chronic liver diseases, including cirrhosis, hepatitis B infection, hepatitis C infection, and other chronic hepatitis. All comorbidities were diagnosed with ICD-9 codes. The accuracy of ICD-9 codes has been examined in previous studies (41–56).

### Major Outcome

The major outcome was a new diagnosis of pulmonary TB (ICD-9 codes 010, 011, 012, and 018) during the follow-up period. All study subjects were followed up with until they were diagnosed with pulmonary TB or to the end of 2013.

### Statistical Analysis

The distributions of sex, age, and comorbidities were compared between the depression and non-depression groups using the chi-square and Fisher-exact test for categorized variables, and the *t*-test for continuous variables. The incidence of pulmonary TB was estimated as the number of pulmonary TB events identified during the follow-up period, divided by the total follow-up person-years for each group. Initially, all variables were included in a univariable model. Next, variables found to be statistically significant in the univariable model were further included in the multivariable model. The multivariable Cox proportional hazards regression model was used to estimate the hazard ratio (HR) and 95% confidence interval (CI) for the risk of pulmonary TB associated with depression and other comorbidities. All analyses were performed using the SAS 9.2 (SAS Institute Inc., Carey, NC, USA). The results were considered statistically significant when two-tailed *P* values were less than 0.05.

## Results

### Baseline Characteristics of the Study Population

Baseline characteristics of the study population have been depicted in Table 1. There were 34,765 subjects in the depression group and 138,187 subjects in the non-depression group, with a similar distribution of sex. The mean ages (SD) of the study subjects were 47.9 (16.5) and 47.6 (16.6) years for the depression and non-depression group, respectively. The depression group had higher proportion of asbestosis, CKD, HIV infection, gastrectomy, pneumoconiosis, and splenectomy than that in the non-depression group (chi-square test, *P* < 0.05 for all).

**Table 1:** Baseline characteristics between depression group and non-depression group.

	Non-depression (*N* = 138,187)		Depression (*N* = 34,765)		
		
Characteristic	*n*	%	*n*	%	*P* value^a^
**Sex**					0.96
Female	52,072	37.7	13,105	37.7	
Male	86,115	62.3	21,660	62.3	
**Age group (years)**					0.61
20–39	49,893	36.1	12,453	35.8	
40–64	62,652	45.3	15,834	45.6	
65–84	25,642	18.6	6,478	18.6	
Age (years), mean (SD)^c^	47.6	(16.6)	47.9	(16.5)	0.002
Follow-up period (years), mean (SD)^c^	8.30	(3.37)	8.21	(3.41)	<0.001
**Baseline comorbidities**					
Alcohol-related disease	9,632	6.97	2,516	7.24	0.08
Asbestosis^b^	8	0.01	6	0.02	0.03
Chronic kidney disease	3,076	2.23	839	2.41	0.04
Chronic liver disease	24,786	17.9	6,314	18.2	0.33
Chronic obstructive pulmonary disease	16,874	12.2	4,330	12.5	0.21
Diabetes mellitus	8,886	6.43	2,304	6.63	0.18
Human immunodeficiency virus infection	140	0.10	52	0.15	0.02
Gastrectomy	273	0.20	109	0.31	0.001
Pneumoconiosis	666	0.48	212	0.61	0.003
Splenectomy	60	0.04	27	0.08	0.01

*Data are presented as the number of subjects in each group with percentages given in parentheses, or mean with SD given in parentheses*.

*^a^Chi-square test, ^b^Fisher-exact test, and ^c^t-test comparing subjects with and without depression*.

### Incidence of Pulmonary TB in the Study Population Stratified by Sex and Age

**Figure 1:**
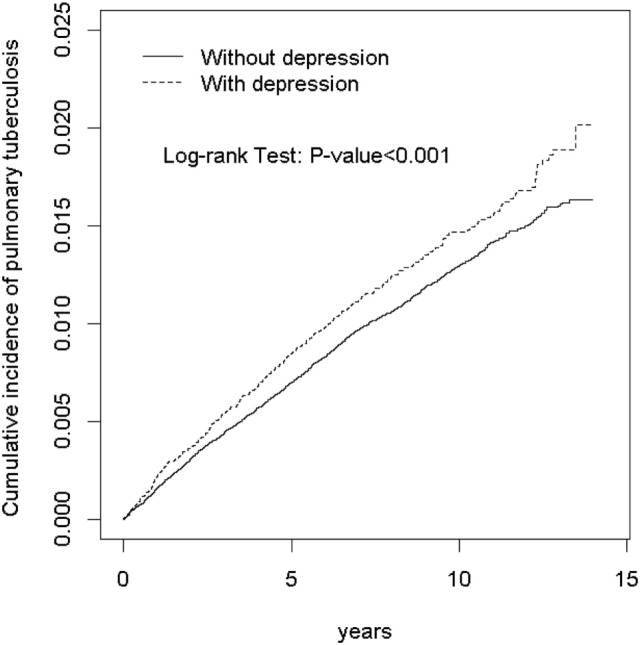
Kaplan–Meier model disclosed that the depression group had a higher cumulative incidence of pulmonary tuberculosis than the non-depression group (1.68 vs. 1.50% at the end of follow-up; *P* < 0.001).

The overall incidence of pulmonary TB was 1.16-fold greater in the depression group than that in the non-depression group (1.52 vs. 1.31 per 1,000 person-years, 95% CI 1.12, 1.21; Table 2). The incidence of pulmonary TB, as stratified by sex and age, was also higher in the depression group than in the non-depression group. The depression group with ages from 65 to 84 years had the highest incidence of pulmonary TB (4.88 per 1,000 person-years). The Kaplan–Meier model revealed that the depression group had a higher cumulative incidence of pulmonary TB than the non-depression group (1.68 vs. 1.50% at the end of follow-up; *P* < 0.001; Figure 1).

**Table 2:** Incidence of pulmonary tuberculosis estimated by sex and age between depression group and non-depression group.

	Non-depression				Depression				
Variable	*N*	Event	Person-years	Incidence^a^	*N*	Event	Person-years	Incidence^a^	IRR^b^ [95% confidence interval (CI)]
All	138,187	1504	1146,960	1.31	34,765	435	285,537	1.52	1.16 (1.12,1.21)
**Sex**									
Female	52,072	569	727,530	0.78	13,105	171	181,913	0.94	1.20 (1.14, 1.26)
Male	86,115	935	419,431	2.23	21,660	264	103,625	2.55	1.14 (1.08, 1.21)
**Age group (years)**									
20–39	49,893	150	433,734	0.35	12,453	63	107,501	0.59	1.69 (1.59, 1.81)
40–64	62,652	528	531,375	0.99	15,834	151	132,729	1.14	1.14 (1.08, 1.21)
65–84	25,642	826	181,851	4.54	6,478	221	45,307	4.88	1.07 (0.99, 1.16)

*^a^Incidence: per 1,000 person-years*.

*^b^IRR (incidence rate ratio): depression vs. non-depression (95% CI)*.

### Pulmonary TB Associated With Depression

We then examined the risk of pulmonary TB associated with depression. Variables found to be statistically significant in the univariable model were further examined in the multivariable model. After adjusting for covariables, the multivariable Cox proportional hazards regression analysis revealed that the adjusted HR for pulmonary TB was 1.15 for the depression group (95% CI 1.03, 1.28) compared with the non-depression group (Table 3).

**Table 3:** Cox model measured hazard ratio (HR) and 95% confidence interval (CI) of pulmonary tuberculosis (TB) associated with depression and comorbidities.

	Crude	Adjusted^a^
	
Variable	HR (95% CI)	HR (95% CI)
Sex (male vs. female)	2.81 (2.56, 3.08)	2.37 (2.16, 2.61)
Age (per 1 year)	1.07 (1.06, 1.07)	1.06 (1.05, 1.06)
Depression	1.16 (1.04, 1.29)	1.15 (1.03, 1.28)
**Baseline comorbidities (yes vs. no)**		
Alcohol-related disease	1.68 (1.44, 1.95)	1.85 (1.58, 2.16)
Chronic kidney disease (CKD)	2.99 (2.44, 3.66)	1.19 (0.97, 1.46)
Chronic liver disease	1.33 (1.19, 1.48)	0.95 (0.85, 1.07)
Chronic obstructive pulmonary disease (COPD)	4.18 (3.80, 4.59)	1.66 (1.50, 1.84)
Diabetes mellitus (DM)	3.29 (2.91, 3.71)	1.55 (1.37, 1.75)
Human immunodeficiency virus (HIV) infection	3.67 (1.65, 8.18)	6.93 (3.10, 15.5)
Gastrectomy	2.76 (1.48, 5.14)	1.18 (0.63, 2.20)
Pneumoconiosis	4.75 (3.43, 6.58)	1.80 (1.29, 2.49)

*^a^Variables found to be statistically significant in the univariable model were further examined in the multivariable model*.

*Adjusted for sex, age, alcohol-related disease, CKD, chronic liver disease, COPD, DM, HIV infection, gastrectomy, and pneumoconiosis*.

*There was no event of pulmonary TB among subjects with asbestosis, or splenectomy*.

### Risk of Pulmonary TB Stratified by Depression and Comorbidities

We also analyzed the risk of pulmonary TB stratified by depression and comorbidities. To reduce the potential confounding effects of comorbidities, we analyzed subjects without depression and without comorbidities. The adjusted HR of pulmonary TB was 1.37 for subjects with depression and without comorbidities (95% CI 1.17, 1.62; Table 4). This finding indicates that even in the absence of comorbidities, depression may have a unique role on the risk of pulmonary TB.

**Table 4:** Risk of pulmonary tuberculosis stratified by depression and comorbidities.

Variable		Event	Person-years	Incidence^a^	Adjusted hazard ratio^b^ (95% CI)
Non-depression	No comorbidity^c^	574	781086	0.73	1 (reference)
Non-depression	Any comorbidity^c^	930	365874	2.54	1.82 (1.63, 2.03)
Depression	No comorbidity^c^	194	193431	1.00	1.37 (1.17, 1.62)
Depression	Any comorbidity^c^	241	92107	2.62	1.87 (1.60, 2.17)

*^a^Incidence rate: per 1,000 person-years*.

*^b^Adjusted for sex and age*.

*^c^Comorbidities including alcohol-related disease, chronic kidney disease, chronic liver disease, chronic obstructive pulmonary disease, diabetes mellitus, human immunodeficiency virus infection, gastrectomy, and pneumoconiosis*.

## Discussion

In this study, we examined the association between depression and pulmonary TB, including relative comorbidities. We found that the mean age of depression group was higher than the non-depression group. In addition, the depression group showed a higher proportion of asbestosis, CKD, HIV infection, gastrectomy, pneumoconiosis, and splenectomy than the non-depression group. By the way, the comorbidities listed in our study might indicate the common ones of Taiwanese, not represent of all ethical groups worldwide. Race and ethnicity may shape the links between comorbid psychiatric disorders and chronic medical conditions. So, we suggest that future studies should put emphasis on the understanding why associations between physical and mental health vary among race and ethnic groups (57). A possible explanation is the lower immune status in the elderly. It has been previously shown that the decreased immune function associated with depression is related to increased susceptibility to immune-mediated diseases, such as cancer, infectious diseases, and autoimmune diseases (58). Furthermore, previous articles have revealed that diabetes, splenectomy, appendectomy, and gastrectomy correlate with the increasing risk of pulmonary TB in Taiwan, compatible with the results of our study (3, 48, 59, 60).

We show that the adjusted HR of pulmonary TB is 1.15 for the depression group (95% CI 1.03, 1.28) compared with the non-depression group. Although previous studies have shown definite association between them worldwide (8, 20, 22), including studies published in Nigeria 8 years ago (61, 62), there were no related studies exploring the relationship between depression and TB in Taiwan. Unlike previous similar studies worldwide, we tried to explain two-way causative relationship between TB and depression. Patients with TB were usually considered related to higher frequency depression due to several reasons mentioned below. They usually isolated from their families, less free due to the complication rate of anti-TB medication and might experience unfair viewpoints form society (6, 19). Furthermore, during the treatment course, they always meet difficulties in financial aspect (63). On the contrary, TB is considered to be more common among individuals with mood disorders as opposed to those with other psychiatric illnesses.

Some past literature revealed that depression patients were more prone to TB infection, the same result in our study, since they are easily to be exposed to risk factors for the disease (8, 64). The major risk factors was discussed in previous literature, such as homeless (65), emigration from a country in which TB is endemic (22). Other minor ones, male gender, drug and alcohol abuser, and previous psychiatric hospitalizations were also noted in published articles (8), which failed to comply with routine TB treatment course, thus result in poor compliance and higher frequency for TB. Although we discussed mutual causative consequences profound, unfortunately, we still could not provide accurate and enough evidence that most risk factors mentioned above whether or not enrolled in our study due to innate limitation. Otherwise, in our study, potential bias and selection bias were still inevitable, future studies should add consideration that the prevalence and incidence of depression or pulmonary TB correlates with severity and duration for increasing the accuracy of studies, similar to previous review article (19, 66). Most of the studies do not mention how to be mindful of the clinical manifestation of depression in the earliest stage of TB and the mutual causative consequences. Thus, we used a large scale, long-term cohort study for exploring the link between depression and the risk of TB.

We hypothesized that lower immune response status may be the key point to understand the association between depression and pulmonary TB. Depression is usually found in elderly population, accompanied with dysphoria, insomnia, and bad mood along with poor appetite (67, 68). Exposure to these morbidities for a long time may result in incomplete and non-comprehensive immunity for protection against micro-organisms, including *Mycobacterium tuberculosis*. In addition, poor appetite and depression results in malnutrition and cachexia. Malnutrition profoundly affects cell-mediated immunity, which has been shown to be the principle host defense against TB (69) in humans and experimental animals. It was also shown by Trenton et al. that TB tends to be isolated in the lungs or other single sites in the hosts with more competent immunity. If the immune system is less robust, the disease tends to proliferate to extrapulmonary sites, including kidneys, bones, or meninges, which may result in subsequent infections of TB (8).

Another possible explanation may be the decreased treatment compliance of pulmonary TB. A large scale retrospective cohort study revealed that patients with TB have a high rate of relapse due to poor medication compliance, psychiatric disorders, alcoholism, and drug addiction (70). Some other psychological issues, such as stigma, isolation, social or family support, and helplessness were also mentioned in previous literature, thus lowered the treatment compliance (19). Patients with depression are usually in a bad mood, easily forgetting scheduled plans, including routine medication for pulmonary TB. Therefore, it may be important to assume that prevention and prompt treatment of psychological disorders in TB patients may be helpful to increase treatment compliance and reduce the relapse of TB.

Finally, the adjusted HR of pulmonary TB was 1.37 for subjects with depression alone and without comorbidities (95% CI 1.17, 1.62; Table 4). This finding shows that depression has an independent role in the risk of pulmonary TB. Thus, general physicians and psychiatrists should pay more attention in evaluating pulmonary TB along with the clinical manifestation of depression.

### Limitation

First, the insurance claims that the data do not provide definite measurement for the clinical diagnosis of depression, such as by DSM-V, and we were unable to evaluate depression by the coding and clinical condition assessment of physicians. More accurate tools for the diagnosis of pulmonary TB, such as chest radiographic films or sputum culture, are necessary. Therefore, the actual number of pulmonary TB in depression population may be underestimated or overestimated. Second, as the diagnosis of depression and pulmonary TB requires long-term observation for its clinical manifestation, the shorter observation period in our study may have been insufficient to estimate the pulmonary TB risk compared with a full course of natural history in clinics or hospitals. The associated variable measures may thus be underestimated. Also, this study included comorbidity information at the baseline, prior to the date of establishing the study cohorts. Thus, comorbidities developed during the follow-up period may vary leading to variabilities in the estimation of pulmonary TB risk. Finally, we found no literature for herd immunity of TB in Taiwan, and socioeconomic status level (6) was not enrolled in our study due to study design and innate limitation, might distortion.

### Strength

One of the primary strength of this study is the set of ICD-9 codes, which has been previously validated in published studies (41–56). Also, the long-term observation period from 2000 to 2012 allowed for more credibility compared with other similar studies to propose physical mechanisms and plausible hypotheses. Finally and the most important, we tried explained mutual biological and psychological mechanism between depression and pulmonary TB.

## Conclusion

We evaluated the risk of pulmonary TB in association with depression and several medical comorbidities in the Taiwanese population using representative population-based data. We demonstrated that patients with depression are at a significantly higher risk of pulmonary TB than those without depression. Other comorbidities may also interact with depression and have a synergistic effect on the risk of pulmonary TB. As depression and pulmonary TB are popular disorders, they have been studied in many other countries for the past few decades. Further studies are needed for patients with depression not only to prevent its clinical exacerbation but also to decrease the possibility of pulmonary TB infection before initialization of depression. The understanding of comorbidities and other psychological factors may yield important preventive and detective methods for reducing the pulmonary TB infection rate. We hope that this study will provide information for earlier intervention and prevention of pulmonary TB in patients with depression worldwide.

## Author Contributions

K-CC and K-FL planned and conducted this study. They participated in the data interpretation and revised the article. C-LL conducted the data analysis and revised the article. S-WL planned and conducted this study. He contributed to the conception of the article, initiated the draft of the article, and revised the article, and contributed equally to the article.

## Conflict of Interest Statement

The authors declare that the research was conducted in the absence of any commercial or financial relationships that could be construed as a potential conflict of interest.
